# Estimating fecal output and nutrient digestibility using acid detergent insoluble ash as an intrinsic marker in lactating dairy cows: technical note

**DOI:** 10.5713/ab.25.0130

**Published:** 2025-05-12

**Authors:** Jungeun Kim, Kiyeon Park, Houhua Hu, Chanhee Lee

**Affiliations:** 1 Interdisciplinary Nutrition Program, Department of Animal Sciences, The Ohio State University, Wooster, OH, USA; 2 Department of Animal Sciences, The Ohio State University, Wooster, OH, USA

**Keywords:** Acid Insoluble Ash, Forage, Indigestible Neutral Detergent Fiber

## Abstract

The current study was part of large experiments in a replicated 4×4 Latin square design where 4 dietary treatments (feed supplements) were examined. Fecal samples were obtained from the experiments via total collection (Experiment 1, n = 30; Experiment 2, n = 16). Feed and fecal samples were analyzed for acid detergent insoluble ash (ADIA) and nutrients, and those were used to estimate fecal output (FO) and nutrient digestibilites. Data within each experiment were analyzed using the Mixed procedure of SAS to determine diet, method (actual vs. estimated by ADIA), and their interaction on FO and apparent total tract digestibility of nutrients. A linear regression for FO between actual and estimated by ADIA was also conducted. The recovery of ADIA was 106% in both experiments. The use of ADIA tended to underestimate FO by 5% (Exp. 2, p = 0.09) and overestimate nutrient digestibilities by 2% to 5% (Exp. 1, p≤0.08). The linear regression resulted in the slope of 0.80 (against 1, p = 0.02) and the intercept of 1.93 (against 0, p = 0.21). In conclusion, ADIA is a potential marker for lactating cows in estimating FO when corrected by the factor obtained from the regression analysis.

## INTRODUCTION

Accurate estimation of fecal output (FO) is essential to determine the digestibility of dietary nutrients in animal nutrition. Total fecal collection is the gold standard to obtain FO and subsequently measure the digestibility of dietary nutrients. However, total collection of feces is laborious and time-consuming and may require specially designed stalls. Therefore, it often limits the number of cows in an experiment. As an alternative approach, intrinsic markers have been used to estimate FO and then digestibility in dairy cows. Reliable intrinsic markers should be easily measurable and nonabsorbable and should not disrupt digestion processes in the gut.

Among the intrinsic markers, acid-insoluble ash (AIA) and indigestible neutral detergent fiber (iNDF) are commonly used in ruminant nutrition studies [1,2]. The advantage of AIA is relatively simple for the analytical procedure. However, a large estimation error was reported in high grain diets [3], suggesting that AIA may not be suitable for all types of diets. The iNDF has been considered a marker that is more reliable than AIA according to comparison studies [4,5]. However, an error still exists in FO and digestibility when estimated using iNDF [4], and the analytical procedure is long and requires cannulated cows [6]. Acid detergent insoluble ash (ADIA) is a potential alternative intrinsic marker and has not received attention like AIA or iNDF. Only a few studies were available in the last 2 decades that examine ADIA as an intrinsic marker to estimate FO. For example, ADIA was examined in dry cows fed burmudagrass hay [7,8] and in steers fed a starter (containing 19% wheat hay) or finishing (no forage) diet [9]. The analytical procedure of ADIA is relatively short and simple, i.e., acid detergent fiber (ADF) followed by ashing of the residual. However, ADIA has not been validated for its suitability in lactating cows which diets are more complicated than small ruminants, beef cattle, or dry cows, i.e., relatively low proportion of forages and various types of ingredients and forages. The objective of this study was to evaluate ADIA on its suitability as an intrinsic marker in estimating FO and apparent total tract digestibility of nutrients in lactating dairy cows.

## MATERIALS AND METHODS

### Study description

The diet and fecal samples were obtained from 2 experiments at the Krauss Dairy Research Center (The Ohio State University, Wooster, OH, USA). Experiment 1 used 8 lactating Holstein cows (214±41 days in milk) in a replicated 4×4 Latin square design with 28-d periods (n = 30, two cows were removed due to health issues). The diets consisted of 50% corn silage, 6% alfalfa silage, and 44% concentrate on a dry matter (DM) basis. The dietary treatments were as follows: a low rumen degradable protein (RDP) diet (9% of dietary DM), the low RDP diet supplemented with iso-butyrate and 2-methyl butyrate (0.216% of dietary DM), a high RDP diet (11% of dietary DM), and the high RDP diet supplemented with iso-butyrate and 2-methyl butyrate. Experiment 2 used 4 lactating Holstein cows (147±63 days in milk) in a 4×4 Latin square design with 19-d periods (n = 16). The diets consisted of 38% corn silage, 19% alfalfa silage, and 43% concentrate on a DM basis. The treatments were as follows: a basal diet (89% Lys requirement), the basal diet with abomasal infusion of 32.1 g/d free Lys, the basal diet topdressed with 122 g/d rumen-protected Lys 1 (65.0 g/d Lys), and the basal diet topdressed with 166 g/d rumen-protected Lys 2 (65.1 g/d Lys). All cows were housed in tie stalls and fed the diets as TMR once a day for ad libitum intake with free access to water.

### Sample collection and analysis

In Experiment 1, feed samples were collected once weekly and composited by periods. Feed samples of Experiment 2 were collected daily during the last 5 days and composited by period. Total collection of feces was conducted for 3 days in Experiment 1 and 5 days in Experiment 2. Feces were subsampled daily for individual cows during total collection and the subsamples were composited by cow and period in proportion to each cow’s daily excretion. The composited feed and fecal samples were dried in an oven at 55°C for 72 h, ground using a Wiley mill (ZM200; Retsch, Haan, Germany) through a 1-mm sieve, and stored at room temperature until further analyses.

An aliquot of the dried and ground feed and fecal samples was dried in an oven at 100°C overnight to determine DM concentration. The samples were ashed in a muffle furnace (BF51800; Thermo Fisher Scientific, Waltham, MA, USA) at 600°C overnight to obtain organic matter (OM) concentration. The concentration of neutral detergent fiber (NDF) in feed and fecal samples weighed out in filter bags (F57; ANKOM Technology, Fairport, NY, USA) and analyzed using ANKOM200 fiber analyzer (ANKOM Technology) with sodium sulfite and heat stable α-amylase according to the manufacturer’s instruction. The N concentration of samples was determined by a combustion method (Flash 2000; Thermo Fisher Scientific). The crude protein (CP) content was calculated as N×6.25.

Another aliquot of feed and fecal samples was assayed for ADIA concentration. Approximately 1.0 g of concentrate and 0.5 g of corn silage, alfalfa silage, and feces were placed into filter bags (F57; ANKOM Technology) and analyzed for ADF using ANKOM200 fiber analyzer (ANKOM Technology) according to the manufacturer’s instruction. The residuals were dried in an air-forced dry oven at 100°C for 2 h and then weighed. The dried bags containing ADF residuals were ashed in a muffle furnace (BF51800; Thermo Fisher Scientific) at 600°C for 8 h. All samples were assayed for ADIA in triplicate. The coefficient of variation (standard deviation÷mean×100) for each sample was calculated for accuracy and precision. If the coefficient of variation was greater than 10%, the samples were reanalyzed until it fell below 10%. The final result of ADIA was used to estimate FO for individual cows.

### Calculations and statistical analysis

FO was estimated using the ADIA concentrations in the diets and feces for individual cows using the following equation: DM intake (DMI)×(ADIA concentration in diet DM)÷(ADIA concentration in fecal DM). Then, the apparent total tract digestibility of DM, OM, NDF, and CP was estimated using actual intake of DM, OM, NDF, and CP and estimated FO of DM, OM, NDF, and CP, respectively. The FO and digestibilities estimated by ADIA were compared with actual FO and digestibilities that were obtained from total collection.

**Figure 1:**
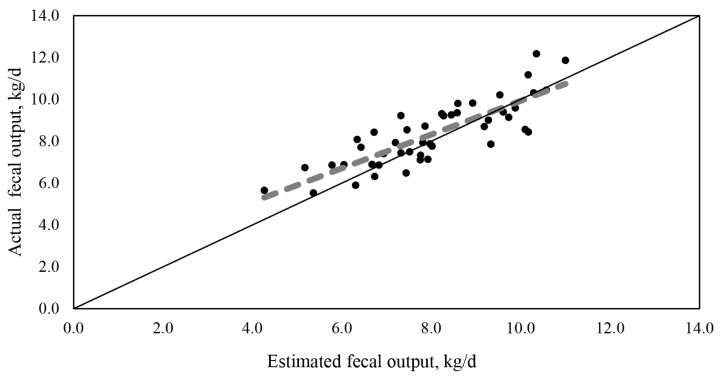
The linear relationship between actual fecal output and fecal output estimated from acid detergent insoluble ash. Observed fecal output = 0.80 (standard error [SE] = 0.082; slope against 1, p = 0.02) × Estimated fecal output+1.93 (SE = 0.679; intercept against 0, p = 0.21). r^2^ = 0.69, concordance correlation coefficient (CCC) = 0.81, and root mean square error (RMSE) = 0.96 (10.6% of the mean).

All data within each experiment were analyzed using the Mixed procedure of SAS (v.9.4; SAS Institute, Cary, NC, USA) to determine whether FO or digestibilities estimated by ADIA were influenced by dietary treatments. The model included fixed effects of dietary treatment, method (estimated by ADIA vs. actual), and interaction between dietary treatment and method, and random effects of period and cow within square. Significance was declared at p<0.05 and a trend was considered at p<0.10. All data from Experiment 1 and 2 were combined for a linear regression analysis between ADIA-estimated and actual FO using the Mixed procedure of SAS with a random effect of experiment. Values of the dependent variable (actual FO) were adjusted (predicted+residual) for the graphical presentation (Figure 1) [10]. The performance of the regression was evaluated using root mean square error (RMSE) [11] and concordance correlation coefficient (CCC) [12]. In addition, the results from the Mixed procedure were restored in the PLM procedure of SAS, and the slope was compared to 1.

## RESULTS

The ADIA concentration was 0.53% and 0.49% for diets and 1.88% and 1.62% for feces in Experiment 1 and 2, respectively (Table 1). The recovery of ADIA in feces was 106.2% and 106.3% for Experiment 1 and 2, respectively. In experiment 1, FO did not differ between actual and ADIA-estimated and was not affected by dietary treatments (Table 2). However, apparent total tract digestibilities of DM, OM, NDF, and CP tended to be greater (p≤0.08) when estimated by ADIA compared with actual. No interaction between dietary treatments and method was observed in Experiment 1. In Experiment 2, FO estimated by ADIA tended to be lower (8.70 vs. 9.07 kg/d; p = 0.09) compared with actual FO. However, the apparent total tract digestbilities of DM, OM, NDF, and CP did not differ between actual and estimated by ADIA. As in Experiment 1, FO was not affected by dietary treatments and no interaction between method and diet was observed in Experiment 2. The linear regression between actual and ADIA-estimated FO from the combined data in Experiment 1 and 2 resulted in the following relationship (Figure 1): actual FO = 0.80(SE = 0.082)×Estimated FO+1.93(SE = 0.679). The slope was different (p = 0.02) from 1, and the intercept was not different from 0. An evaluation of the model indicated r^2^ of 0.69, CCC of 0.81, and RMSE of 0.96 (10.6% of the mean).

## DISCUSSION

The concentration of ADIA in diets varies depending on the ratio of forage to concentrate. Because of relatively high concentration of ADIA in forages versus concentrates [13,14], the greater the forage in a diet, the greater the ADIA concentration in the diet. Although a few studies are available, ADIA was used as an intrinsic marker for the purpose of estimating FO followed by total tract digestibility of nutrients. In most studies, however, diets rich in forages were often used. For example, Undersander et al [15] fed alfalfa hay supplemented with mineral salts to lambs, and Kanani et al [7] used bermudagrass hays with mineral salts for dry cows. In those studies, the recovery of ADIA in feces was on average 105.2% [15] and 103% [7], respectively. According to the recovery rates in those studies, ADIA is likely acceptable as an intrinsic marker to estimate FO when compared with other markers that are widely used (e.g., 104% for AIA or 108% for iNDF [1]). However, ADIA as an internal marker may not be suitable for diets containing low insoluble ash concentrations, e.g., relatively low forage diets [3], because low concentration results in large analytical variation which can cause errors in estimating FO. Therefore, the aim of this study was to validate ADIA as an intrinsic marker to estimate FO and nutrient digestibility in lactating cows fed a diet consisting of forages and concentrate (e.g., about 55:45) which is a typical diet for lactating cows in North America and contains relatively low ADIA compared with a forage-based diet described above.

Dietary concentrations of ADIA (0.53% and 0.49%) in the current study were in the range observed in a previous study where relatively high and low forage-based diets were examined, e.g., 1.6% and 0.3% for starter (35% forage and 65% concentrate on a DM basis) and finishing diets (8% forage and 92% concentrate) of steers, respectively [9]. To be an ideal intrinsic marker, the marker should not be absorbed in the digestive tract, meaning that the amount of the marker consumed via diets should be completely recovered in feces. There are a few studies that evaluated ADIA as an intrinsic marker to estimate FO and nutrient digestibility. In the experiment with steers [9], the recovery of ADIA in feces was 141% and 292% when a starter or finishing diet, respectively, was fed. As a result, the DM digestibility (DMD) estimated by ADIA for the starter diet was similar when compared with that from total collection (72.9% vs. 71.7%) but overestimated by ADIA for the finishing diet (94.5% vs. 82.3%; p<0.05). In a study by Kanani et al [7], dry cows were fed bermudagrass hays (2.0% to 3.2% ADIA on a DM basis) and the recovery in feces was 103%, and the FO and DMD estimated by ADIA were not different from those measured from total collection. When lambs were fed alfalfa hay with trace mineral salts (3.6% to 9.8% of ADIA on a DM basis), the recovery of ADIA was 105%, estimating the DMD being similar to that measured from total collection [15]. According to the studies above, clear overestimation of ADIA recovery and DMD was found only for the finishing diet when ADIA was used as a marker [9]. The authors demonstrated that the overestimation of the recovery and DMD occurred due to additional ADIA consumption from the pen surface or feces being contaminated with soils in an unrestricted condition. In addition, analytical variation was also pointed out because ADIA concentration was low in the finishing diet (i.e., low forage). Indeed, Thonney et al [3] suggested the inadequacy of ADIA as an intrinsic marker to estimate FO and DMD if the concentration in a diet is below 0.75% on a DM basis.

In the current experiment, although ADIA concentrations in the diets were less than 0.75% of dietary DM, the recovery of ADIA observed in the 2 experiments (106%) was reasonable and was similar to those observed in previous studies where forage-based diets were used [7,15]. The recovery was greater than 100% in both experiments, but it was not like the severe overestimation mentioned above from the study with steers that was conducted in a unrestricted condition [9]. As described above, the reasonable recovery in the current study can be explained by the restricted condition under which the current experiments were conducted. Because cows were housed in tiestalls and fecal samples were collected directly into a container without contamination with soils, the severe overestimation of the recovery of ADIA did not occur. However, although it was not severe, the recovery of ADIA in feces was overestimated and consistent in both experiments (106%). The overestimation was evident by a tendency for lower FO in Experiment 2 and greater digestibilities in Exp.1 when estimated by ADIA compared with actual from total collection. Although Experiment 1 and 2 showed no difference in FO and nutrient digestibilities, respectively, between ADIA-estimated and actual, both experiments had a similar underestimation of FO by about 0.3-kg units for FO and overestimation of nutrient digestibilities by 1% to 2%-units. The difference in statistical inference between Experiment 1 and 2 may have occurred due to the number of animals used in the experiments (n = 30 and 16, respectively). Although the overestimation of fecal ADIA excretion was true, the difference in FO and DMD between estimated by ADIA and actual was small (<5%) and no interaction of method by dietary treatments was observed. Therefore, we concluded that ADIA as an intrinsic marker is a valid marker for diets fed to lactating cows (e.g., similar proportions of forage and concentrate on a DM basis) if a correction factor is applied as shown below.

According to the regression analysis, the necessity of correcting FO estimated by ADIA is clear by a factor. According to the linear relationship, although the model showed good CCC and RMSE, the underestimation of FO by ADIA was clear with the slope of 0.80. In addition, within the range of data used in the study, the lower the FO, the greater the underestimation of FO by ADIA, which is difficult to explain. However, it could be possible that there was a source of fecal contamination such as gut secretion of minerals [16] or concrete dusts in the feed bunk or on the floor that may have been introduced into the fecal collection container. If those were the source contaminating feces during the collection period, the concentration and recovery of ADIA in feces might have been impacted more when fecal excretion was small (e.g., dilution). As a result, we obtained the equation in Figure 1, and it can be used to correct the value of FO estimated by ADIA in lactating cows fed a diet containing similar proportions of forage and concentrate (DM basis).

## CONCLUSION

The concentrations of ADIA were 0.53 and 0.49% in the diets used for lactating cows which had similar proportions of forages and concentrate. The average recovery of ADIA was 106% which is acceptable as an intrinsic marker but obviously overestimated. The overestimation of the recovery led to an underestimation of FO and an overestimation of nutrient digestibilities when estimated by ADIA, compared to actual values obtained from total collection. The underestimation of FO increased as FO was decreased according to a linear model. Therefore, ADIA can be a potential intrinsic marker to estimate FO of lactating cows if corrected according to the equation obtained from the linear regression analysis in lactating cows fed a diet containing similar proportions of forage and concentrate on a DM basis.

## Figures and Tables

**Table 1:** The descriptive summary of dietary and fecal acid detergent insoluble ash (ADIA) concentrations and the recovery of ADIA in feces

Item	Experiment 1^1)^ (n = 30)		Experiment 2^2)^ (n = 16)	
	
	Mean	SD	Mean	SD
ADIA concentration (% of DM)				
Diets	0.53	0.007	0.49	0.006
Feces	1.88	0.26	1.62	0.18
ADIA recovery (%)	106.2	14.8	106.3	20.9

1) Diets consisted of 50% corn silage, 6% alfalfa silage, and 44% concentrate and containing 59.6% dry matter, 93.5% organic matter, 29.2% neutral detergent fiber, and 17.7% crude protein on a dry matter basis.

2) Diets consisted of 38% corn silage, 19% alfalfa silage, and 43% concentrate and containing 62.8% dry matter, 93.7% organic matter, 31.4% neutral detergent fiber, and 15.2% crude protein on a dry matter basis.

ADIA, acid detergent insoluble ash; SD, standard deviation; DM, dry matter.

**Table 2:** Estimating the fecal output and dry matter digestibility using acid detergent insoluble ash (ADIA) as an intrinsic marker

Items	Actual	Estimated	SEM	p-values^1)^		

				Method	Diet	Int
	Experiment 1					
Fecal output (kg/d)	7.90	7.63	0.465	0.17	0.38	0.60
Apparent digestibility (%)						
DM	70.3	71.5	0.87	0.06	0.53	0.40
OM	71.2	72.3	0.89	0.07	0.44	0.42
NDF	45.4	47.4	2.07	0.08	0.33	0.43
CP	72.7	73.9	1.04	0.08	<0.01	0.50
	Experiment 2					
Fecal output (kg/d)	9.07	8.70	0.974	0.09	0.38	0.39
Apparent digestibility (%)						
DM	68.2	69.5	1.74	0.15	0.15	0.49
OM	69.1	70.3	1.71	0.15	0.15	0.48
NDF	44.3	46.5	2.85	0.18	0.17	0.53
CP	63.0	64.4	2.25	0.16	0.16	0.53

1) Method, actual (total collection) vs. estimated (ADIA); Diet, dietary treatment effect; Int, interaction between method and diet.

SEM, standard error of the means; DM, dry matter; OM, organic matter; NDF, neutral detergent fiber; CP, crude protein.
